# ANFIS-based forming limit prediction of stainless steel 316 sheet metals

**DOI:** 10.1038/s41598-023-28719-5

**Published:** 2023-02-22

**Authors:** Mingxiang Zhang, Zheng Meng, Morteza Shariati

**Affiliations:** 1 Chongqing Creation Vocational College, Yongchuan, 402160 Chongqing China; 2 College of Applied Technology, Dalian Ocean University, Dalian, 116300 Liaoning China; 3 Faculty of Engineering, Islamic Azad University, North Tehran Branch, Tehran, Iran

**Keywords:** Mechanical engineering, Materials science, Mathematics and computing

## Abstract

Effect of microstructure on the formability of the stainless sheet metals is a major concern for engineers in sheet industries. In the case of austenitic steels, existence of strain-induced martensite (${\alpha }^{^{\prime}}$-martensite) in their micro structure causes considerable hardening and formability reduction. In the present study, we aim to evaluate the formability of AISI 316 steels with different intensities of martensite via experimental and artificial intelligence methods. In the first step, AISI 316 grade steels with 2 mm initial thicknesses are annealed and cold rolled to various thicknesses. Subsequently, the relative area of strain-induced martensite are measured using metallography tests. Formability of the rolled sheets are determined using hemisphere punch test to obtain forming limit diagrams (FLDs). The data obtained from experiments were further utilized to train and validate an artificial neural fuzzy interfere system (ANFIS). After training the ANFIS, predicted major strains by the neural network are compared to a new set experimental results. The results indicate that cold rolling has unfavorable effects on the formability of this type of stainless steels while significantly strengthens the sheets. Moreover, the ANFIS exhibits satisfactory results in comparison to the experimental measurements.

## Introduction

The formability of sheet metals although has been the subject of research articles for decades, is yet an interesting field of study in metallurgy. The new technological instruments and computational models make it easier to find about the underlying factors affecting formability. Most importantly, using crystal plasticity finite element methods (CPFEM) in recent years reveals the significance of microstructure on the forming limits. On the other hand, availability of scanning electron microscope (SEM) and Electron Backscatter Diffraction (EBSD) have aided researchers to observe microstructural activities of the crystalline structures during deformation. Understanding of effects of different phases in metals, grain size and orientation and micro-scale imperfection in the grain level are vital in prediction of formability.

Formability determination itself is challenging procedure since it has been proven that formability is highly path-dependent^1–3^. Therefore, conventional forming limit strains representation could not be reliable in non-proportional loading conditions. On the other hand, the majority of loadings paths in industrial application is categorized as non-proportional. In this regard, conventional hemi-sphere and Marciniak–Kuczynski (M–K) experimental methods should be utilized with caution^4–6^. In recent years, another concept of fracture forming limit diagram (FFLD) has attracted the attention of many engineers in the field of formability. In this concept, the formability of the sheets is predicted using the damage models. In this regard, path independency is intrinsically incorporated in the analyses and the results are in good agreement with non-proportional experimental results^7–9^. Formability in sheet metals has dependency on several parameters and processing history of the sheets and also on the microstructure and phases in metals^10–15^.

Size-dependency is a challenge in incorporation of micro-features on metals. In small-deformation space, the dependency of the vibration and buckling characteristics has proven to be strongly dependent on the length scale of the materials^16–30^. Effects of grain size on the formability have been recognized in industry for a long time. Effects of grain size and thickness on the stretchability of sheet metals were examined using theoretical analysis by Yamaguchi and Mellor^31^. Using Marciniak model, they reported that decrease in the thickness to grain size ratio causes decrease in stretchability of sheets in biaxial stretch loading condition. Experimental results by Wilson et al.^32^ confirm that reduction in the thickness to average grain diameter (t/d) leads to decrease in biaxial stretchability of three different sheet metals with various thicknesses. They concluded that for t/d values less than 20, the prominent strain inhomogeneity and necking are mostly affected by individual grains in the thickness of the sheet. Effect of grain size austenitic stainless steels 304 and 316 on the bulk workability was investigated by Ulvan and Koursaris^33^. They reported that the formability of these metals were not affected by grain size but slight variation in the tensile characteristics were seen. In specific, increase in grain size resulted in decrease the strength measures of these steels. Examination of the effect of dislocation density on the flow stress of nickel metal reveals that irrespective of grain size, it is the dislocation density that determines the flow stress of the metal^34^. Grain interaction and initial orientations also have significant influence on the texture evolution in aluminum as examined by (Becker and Panchanadeeswaran using experimental and crystal plasticity simulations^35^. The numerical results in their analyses were in good harmony with experiments although due to limitations in applying boundary condition some simulation results deviated from experiments. Rolled aluminum sheets manifested different formabilities as detected crystal plasticity simulations and experimental examination^36^. It was shown that although the stress–strain curves of different sheets were almost similar, there were a significant differences in their formability based on the initial textures. Amelirad and Assempour utilized experimental and CPFEM to obtain forming limit curves in austenitic stainless steel sheet metal^37^. Their simulation reveals that increase in grain size shifts forming limit curves upward in FLD. Moreover, grain orientation and morphology effects on the void nucleation were examined by the same authors^38^.

Beside grain morphology and orientation, the state of twinning and second phases are important in austenitic stainless steels. Twinning is the main mechanism of hardening and elongation improvement in the TWIP steels^39^. Hwang^40^ reported that the formability of TWIP steels is not satisfactory despite the adequate tensile responses. However, the effects of strain-induced twinning on the formability of austenitic steel sheets is not recognized well. Mishra et al.^41^ examined austenitic stainless steel to observe twinning generation under different stretching strain paths. They found that twinning could be generated from both sources of decay of annealing twins and new generation of twins. It was observed under biaxial stretching the maximum twins are generated. Moreover, austenite to ${\alpha }^{^{\prime}}$-martensite transformation was observed to be strain path dependent. Hong et al.^42^ explored the effects of strain-induced twinning and martensite in selective laser melted austenitic steel 316 L on the hydrogen embrittlement in a range of temperature. It was observed that based on the temperature value, the hydrogen could results in fracture or enhancing formability of the 316 L steels. Volume of strain-induced martensite under tensile loading condition in various loading rate was measured experimentally by Shen et al.^43^. It was revealed that increase in the tensile strain increases the volume fraction of martensite fractions.

Using artificial intelligence methods in the science and engineering fields are increasing due to their versatility in modeling complex problems without engaging the physical and mathematical foundations of the problem^44–52^. Moradi et al.^44^ utilized machine learning method to optimize the chemical condition leading to producing smaller nano-silica particle. Other chemical properties also affected the nano-scale material properties as investigated in many research articles^53^. Xie et al.^45^ engaged ANFIS to predict formability of plain carbon steel sheet metals under different rolling conditions. Dislocation density in low carbon steels increases extensively due to cold rolling. The mechanism of the hardening and reducing formability is different in the plain carbon steel from austenitic stainless steels. In plain carbon steel no phase transformation occur in the microstructure of the metal. Beside the phase of metals several other microstructural features arising from different processing of heat treatmetnt, cold working, aging affects the ductility, fracture, machinability, etc. of the metals^54–62^. Recently, Chen et al.^63^ considered the effects of cold rolling on the formability of 304 L steels. They considered only phenomenological observations in the experimental tests to train a neural network on for prediction of the formability. Indeed, several factors combines in reduction of stretchability of sheets in the case of austenitic stainless steels. Lu et al.^64^ utilized ANFIS to observe effects of different parameters on the hole expansion process.

As discussed briefly in the above review, effect of the microstructure on the forming limit diagrams are scarcely addressed in the literature. On the other hand, the microstructural features to be considered are numerous. Therefore, incorporation of all microstructural factors is hardly possible in analytical methods. In this sense, using artificial intelligence could be helpful. In this regard, effect of an aspect of microstructural factor, namely existence of stress-induced martensite, on the formability of the stainless sheet metals is investigated in the present study. This study contrasts from other AI studies on the formability with its focus on the microstructural features and not only on the experimental FLD curves. We aim to evaluate the formability of 316 steels with different levels of martensite using experimental and artificial intelligence methods. At the first step, 316 steels with 2 mm initial thickness are annealed and cold rolled to various thicknesses. Afterwards, the relative area of martensite are measured using metallography tests. Formability of the rolled sheets are determined using hemisphere punch test to obtain forming limit diagrams (FLDs). The data obtained from are further utilized to train and validate an artificial neural fuzzy interfere system (ANFIS). After, training the ANFIS, the predictions of the neural network are compared to a new set experimental results.

## Experimental setup

### Heat treatment and rolling of sheets

The sheet metal used in the current study in austenitic stainless steel 316 with the chemical composition presented in Table 1 with initial thickness of 1.5 mm. An annealing process at 1050 °C for 1 h followed by water quenching was conducted to remove any residual stress in the sheet and to obtain a uniform micro structure.

**Table 1:** Measured chemical composition of 316 sheet.

C	Si	S	P	Mn	Ni	Cr	Mo	V	Ti	Cu	W	Fe
0.074	0.256	0.006	0.032	1.80	10.70	16.30	2.15	0.053	0.005	0.254	0.071	Rem.

### Metallography test

**Figure 1:**
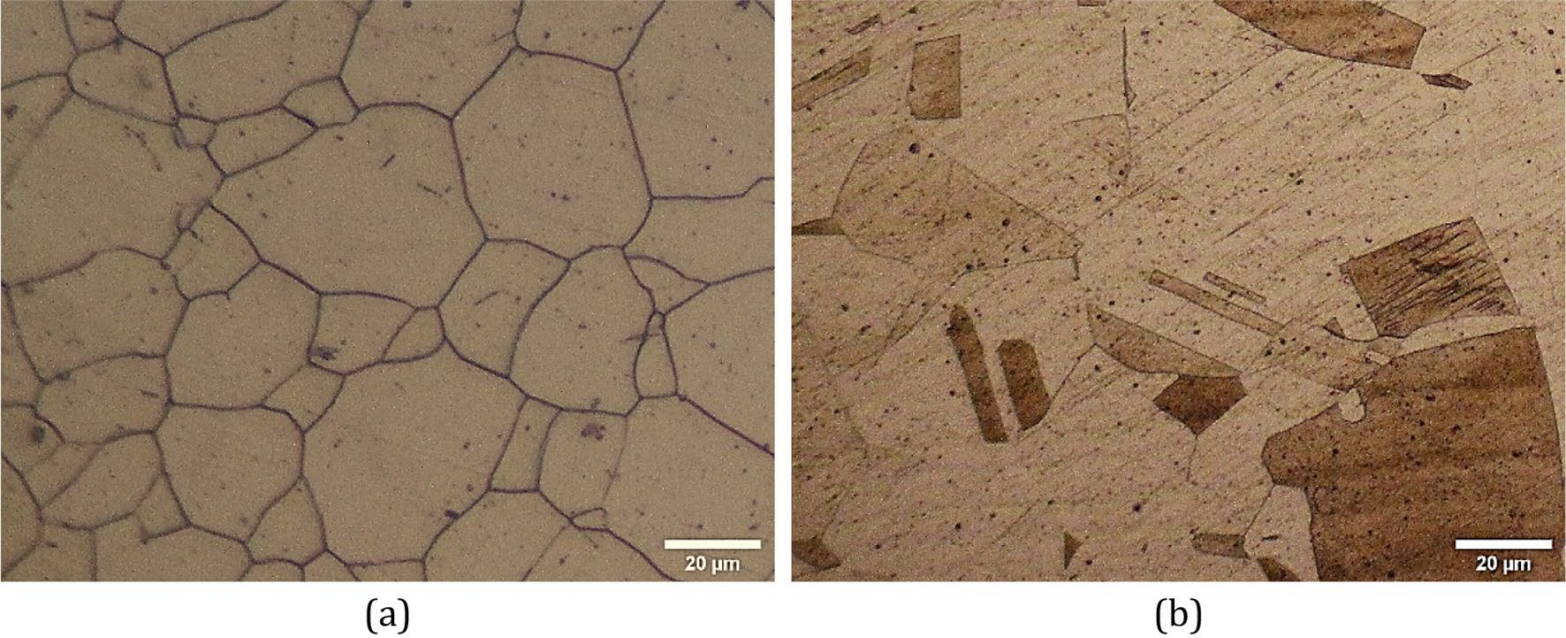
Microstructure of 316 sheet metal after annealing in as received condition as revealed by different etchants, (**a**) 200x, 60% ${\mathrm{HNO}}_{3}$ in distilled water at 1.5 V for 120 s, and (**b**) 200x, acetic glyceregia.

The microstructure of the austenitic steels could be revealed using several etchants. One of the best etchants are 60% nitric acid in distilled water at 1 V direct current for 120 s^38^. However, this etchant only reveals grain boundaries and the twin boundaries could not be recognized as could be observed in Fig. 1a. One another etchants is acetic glyceregia in which the twin boundaries are well revealed but grain boundaries are not revealed very well as seen in Fig. 1b. Moreover, after transforming metastable austenitic phase to ${\alpha }^{^{\prime}}$-martensite phase, the martensite could be revealed using acetic glyceregia etchants which is of interest in the current study.

## Cold rolling process

**Figure 2:**
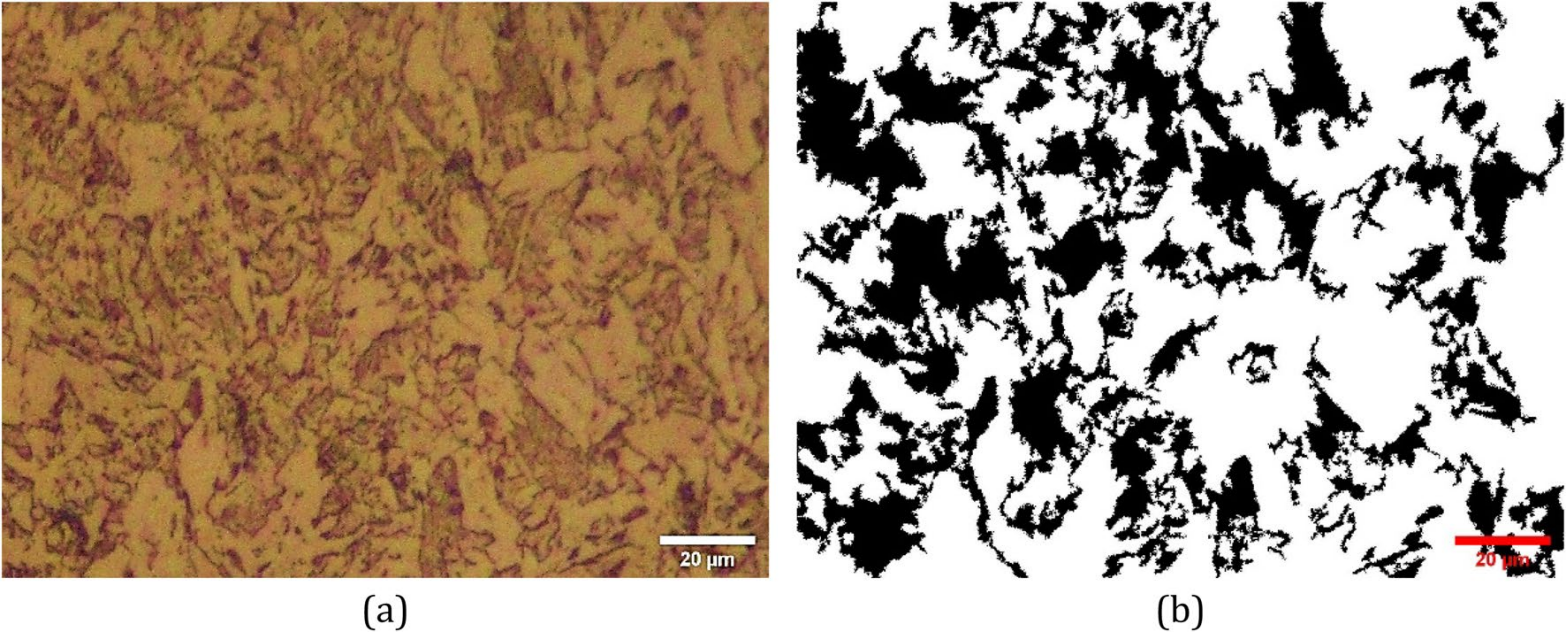
Microstructure of 316 L sheet after being rolled to 50% thickness reduction as viewed from normal to sheet plane direction, 200x, acetic glyceregia.

The annealed sheets were cut to 11 cm width and 1 m long for the rolling purpose. The cold rolling apparatus had two symmetric rolls with 140 mm diameters. The cold rolling process induces the transformation of austenite to strain-induced martensite in 316 stainless steel. We are seeking the ratio of martensite phase to austenite phase after cold rolling to different thicknesses. A sample of the rolled sheet microstructure is shown in Fig. 2. Figure 2a demonstrates the metallography image of the rolled sample as seen from the normal to sheet direction. In Fig. 2b, the portion of the martensite is contrasted with black color using ImageJ software^65^. Using the tools in this open-source software it is possible to measure the area of the martensite portion. A detailed fraction of martensite to austenite phase after rolling to various thickness reductions are provided in Table 2.

**Table 2:** Different thickness reduction the 316 stainless steel sheet in cold rolling process and their respective martensite fraction measure.

Thickness reduction (%)	10	21	29	40.5	50
Mean martensite fraction (%)	7.8	15.4	18.3	25.6	28.7
Standard deviation (%)	2.5	3.8	2.4	4.1	3.4

**Figure 3:**
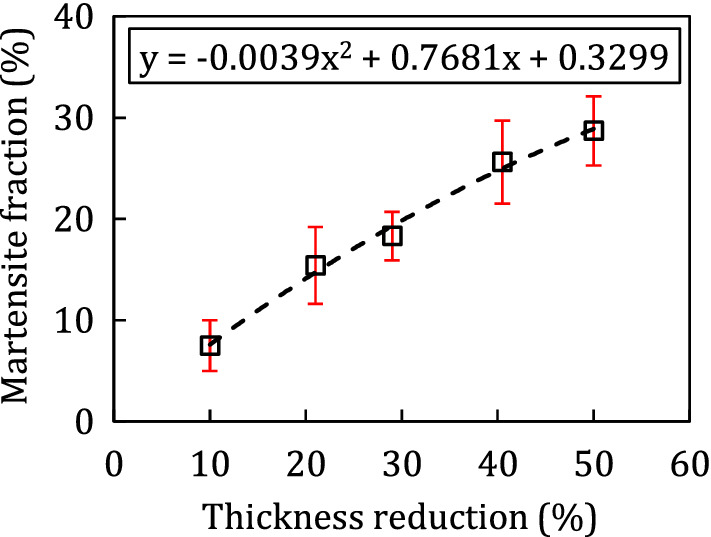
Change in the martensite fraction as finction of thickness reduction in cold rollign process for initrially annealed 316 sheets.

The values provided in Table 2 are obtained by averaging martensite fraction measured from three pictures from the different locations of same metallographic sample. Moreover, a quadratic fitting curve is displayed in Fig. 3 to have a closer insight into the effect of cold rolling on the martensite. It is seen that almost a linear correlation is maintained between martensite fraction and cold rolling thickness reduction. However, a quadratic relation has better representation for this relationship.

### Forming limit measurement

**Figure 4:**
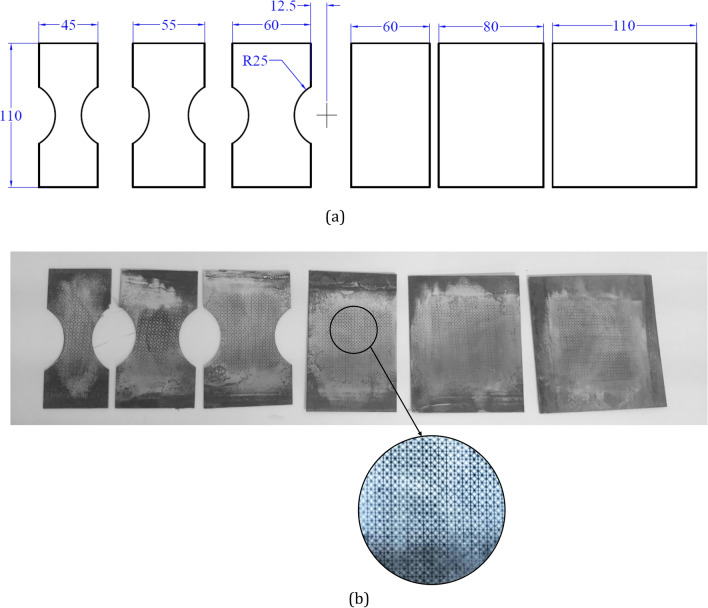
Nakazima’s forming limit samples dimensions and cut sheets. (**a**) dimensions, (**b**) cut and marked samples.

The forming limit evaluations follow a routine procedure using hemisphere punch test^37,38,45,66^. In total, six samples with the dimensions given in Fig. 4a are prepared by laser cutting method as one set of experiment samples. For each martensite fraction condition, three sets of test samples are prepared and tested. Figure 4b shows the cut, polished and marked samples.

**Figure 5:**
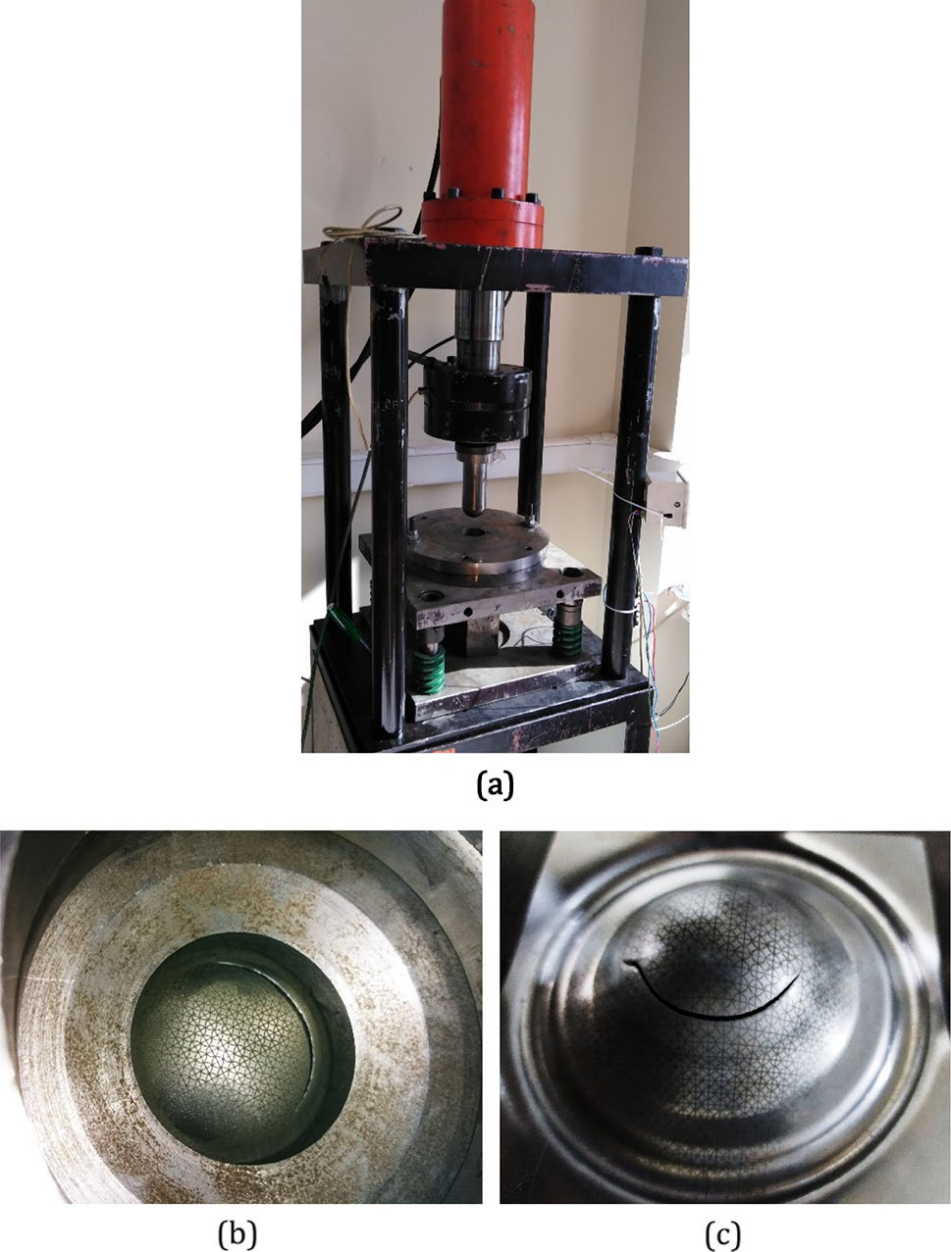
Forming limit determination using hemisphere punch test, (**a**) Test aparatus (**b**) Sample sheet in aparatus in time of fracture, (**c**) The same sample after test.

The hemisphere punch test are conducted using hydraulic press with 2 mm/sec displacement rate is used. The contact surfaces of punch and sheet are lubricated sufficiently to minimize the effect of friction on the forming limit values. The tests were continued until an apparent necking or fracture was observed in the sample. In Fig. 5, a fractured sample in apparatus and after test are shown.

## Neuro-fuzzy system in predicting FLD method

The neural-fuzzy system developed by Jang^67^ is a suitable tool in predicting forming limit curves of sheet metals. This type of artificial neural networks incorporates the effects of parameters having fuzzy descriptions. It means that they could take any real value in their domain. This type of values is further categorized based on their value. Each category would affected by its respective rule. For example, the temperature value could be any real number, Based on its value it could be categorized as cold, moderate, warm and hot temperature. In this regard, the rule for cold temperature is, for example, “wear a jacket” and for the warm temperature is “A T-shirt is enough”. In the fuzzy logic itself, the output results are evaluated for its accuracy and reliability. Incorporating neural network system with fuzzy logic make it possible to ensure the ANFIS provides reliable results.

**Figure 6:**
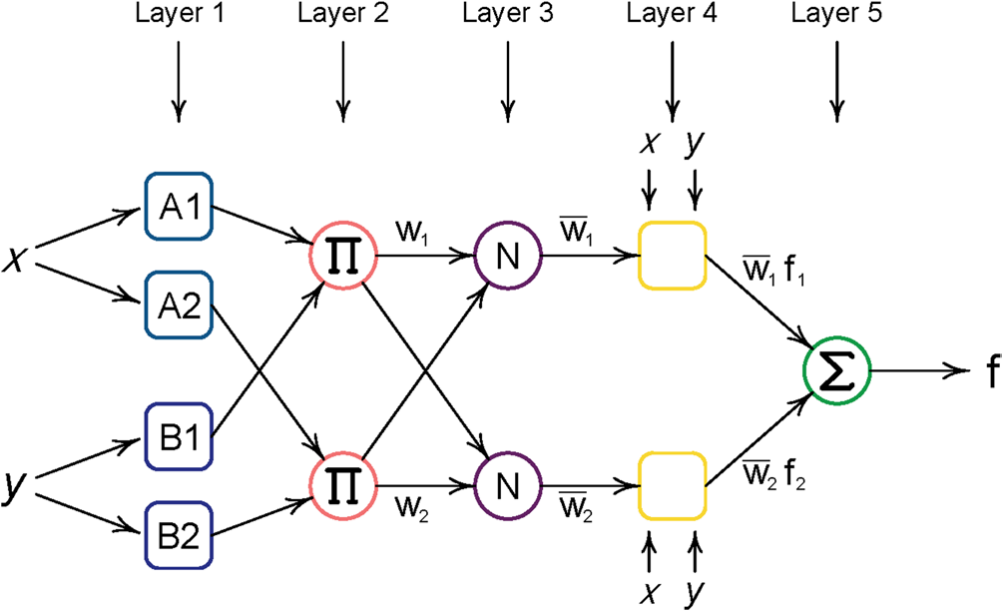
Schematic of ANFIS layers for two inputs and one outputs^67^.

A simple neural-fuzzy network is depicted in Fig. 6 as provided by Jang^67^. As seen in this figure, the network accepts two inputs which in the case of our study the inputs are martensite fraction in microstructure and minor strain value. In the first layer of analysis the fuzzification of the input values is performed using fuzzy rules and membership functions (MFs):

1 $${\mu }_{{A}_{i}}\left(X\right), {\mu }_{{B}_{i}}\left(Y\right),$$

for $i=1, 2$, since the input data assumed to have two description categories. The MFs could take any form of triangle, trapezoidal, Gaussian or any other forms.

**Figure 7:**
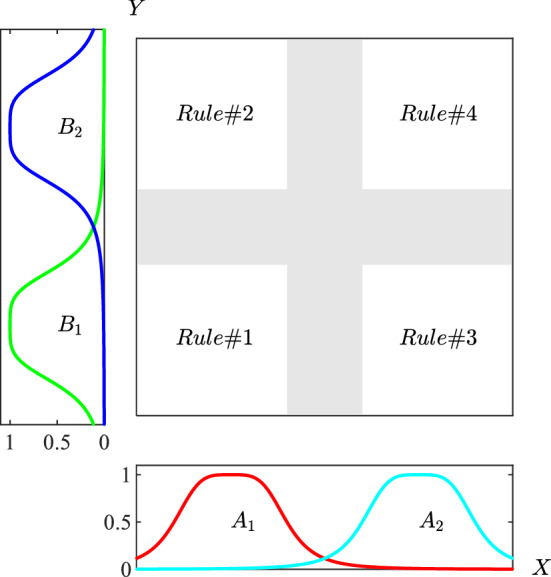
Fuzzy subspaces for ANFIS with two inputs and four rules.

Based on the categories of ${A}_{i}$ and ${B}_{i}$ and their MF values some rules are adopted in the layer 2 as depicted in Fig. 7. In this layers, effects of different inputs are combined in a certain way. Here, the following rule are used to combine the effects of martensite fraction and minor strain values:

2 $${w}_{i}={\mu }_{{A}_{i}}\left(X\right)\times {\mu }_{{B}_{i}}\left(Y\right).$$

The output of this layer, ${w}_{i}$, are called firing strength. These firing strengths are normalized following the below relation in Layer 3:

3 $${\overline{w} }_{i}=\frac{{w}_{i}}{\sum_{k}{w}_{k}}.$$

In Layer 4, Takagi and Sugeno’s rule^67,68^ is incorporated in the calculations to consider the effects of initial values of input parameters. The following relation is held in this layer:

4 $${f}_{i}={p}_{i}X+{q}_{i}Y+{r}_{i}.$$

The obtained ${f}_{i}$ were affected by the normalized values in layers to give the final output which is the value of major strain:

5 $$f=\sum_{k=1}^{NR}{\overline{w} }_{k}{f}_{k},$$

where $NR$ denotes number of rules. The role of the neural network here is to use its internal optimization algorithms to adjust unknown parameters of the network. The unknown parameter are the *consequent parameters* $\left\{{p}_{i}, {q}_{i}, {r}_{i}\right\}$ and parameters related to MFs which are considered to be generalized bell-shape functions:

6 $$f\left(x;a,b,c\right)=\frac{1}{1+{\left|\frac{x-c}{a}\right|}^{2b}}.$$

### Using ANFIS in determining FLD

Forming limit diagrams are dependent on many parameters from chemical composition to history of deformation in the sheet metals. Some parameters are simple to evaluate including the tensile test parameters and some others take more complicated procedure like metallography or determination of residual stress. In most cases, it is beneficial to perform forming limit test on each batch of sheets. However, sometimes the results other tests could be utilized in approximating forming limits. For an account, several studies have utilized tensile testing results to determine the formability of sheets^69–72^. Other studies incorporated more parameters like thickness and grain size into account in their analyses^31,73–77^. However, incorporating all effective parameters are not computationally beneficial. Therefore, using ANFIS models could be a reasonable approach to these problems^45,63^.

In this work, the effect of martensite fraction value on the forming limit diagram of austenitic steel 316 sheets. In this regard, a data set is prepared using experimental tests. The designed system has two input variables: martensite fraction as measured in metallography tests and minor engineering strain range. The output is taken to be major engineering strain of the forming limit curve. The categories of the martensite fraction is three categories of low, medium and high fractions. With low, it is meant that the martensite fraction is below 10%. In the medium condition, the martensite fraction falls between 10 and 20%. The high value of the martensite is regarded as fractions above 20%. In addition, the minor strain has three distinct categories of between − 5 and 5% near vertical axis for determination of FLD0. The positive and negative ranges are two other categories.

## Results

### Forming limit results

**Figure 8:**
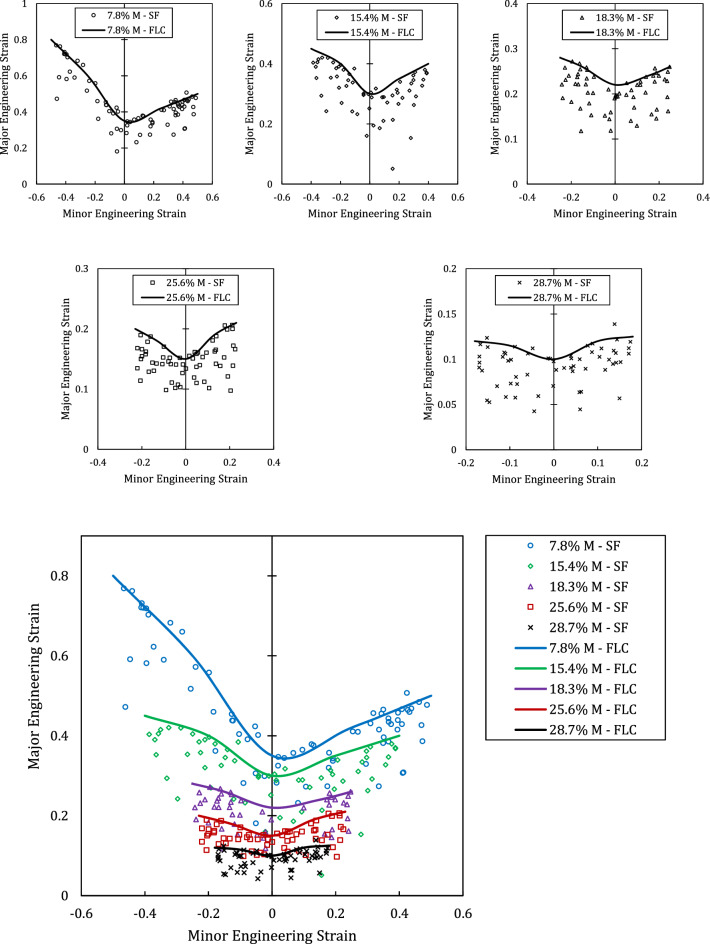
Foming limit curves of 316. Effect of martensite fraction on the formability of austenitic steel sheets. (*SF* safe points, *FLC* forming limit curve, *M* martensite).

The results of the hemisphere tests are presented in Fig. 8. This figure contains 6 forming limit diagram, 5 of them are the FLDs of a single rolled sheets. The safe points and their upper bound curve, forming limit curve (FLC), are presented. In the last graph, all the FLCs are compared. As seen from the last graph, increase in the martensite fraction of the austenitic steel 316 reduces the formability of the sheet metal. On the other hand, increase in the martensite fraction gradually shapes the FLCs to a symmetric curve about vertical axis. In the last two graphs, the right side of the curves are slightly higher than left side meaning that formability in the biaxial stretch conditions are higher than uniaxial stretch loading. Moreover, both minor and major engineering strains at before necking reduce with increase in the martensite fraction.

### ANFIS results

**Figure 9:**
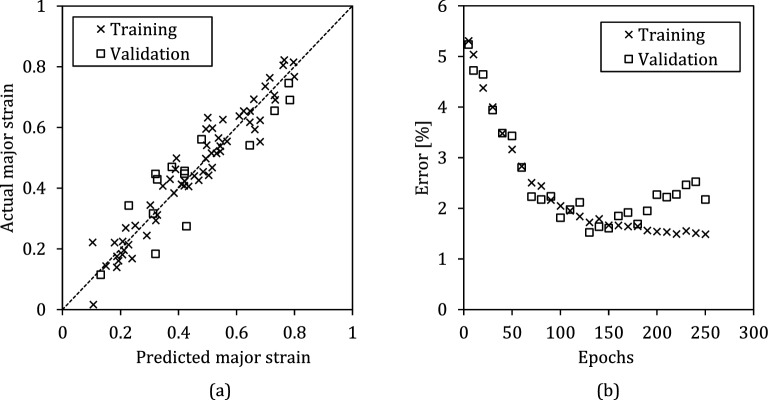
(**a**) correlation between predicted and actual values after training process, (**b**) Error between predicted and actual values of major engineering strain on FLC for both training and validation process.

Training of the neural network is implemented using 60 set of the experimental results of 7.8, 18.3 and 28.7% martensite fractions. The data set from 15.4% martensite are reserved for validation process and 25.6% for testing process. The error after 150 epoch was around 1.5%. The correlation between provided actual output (${\epsilon }_{1}$, major engineering strain) for both training and testing are depicted in Fig. 9. As observed, the trained NFS satisfactorily predicts ${\epsilon }_{1}$ for the sheet metals.

**Figure 10:**
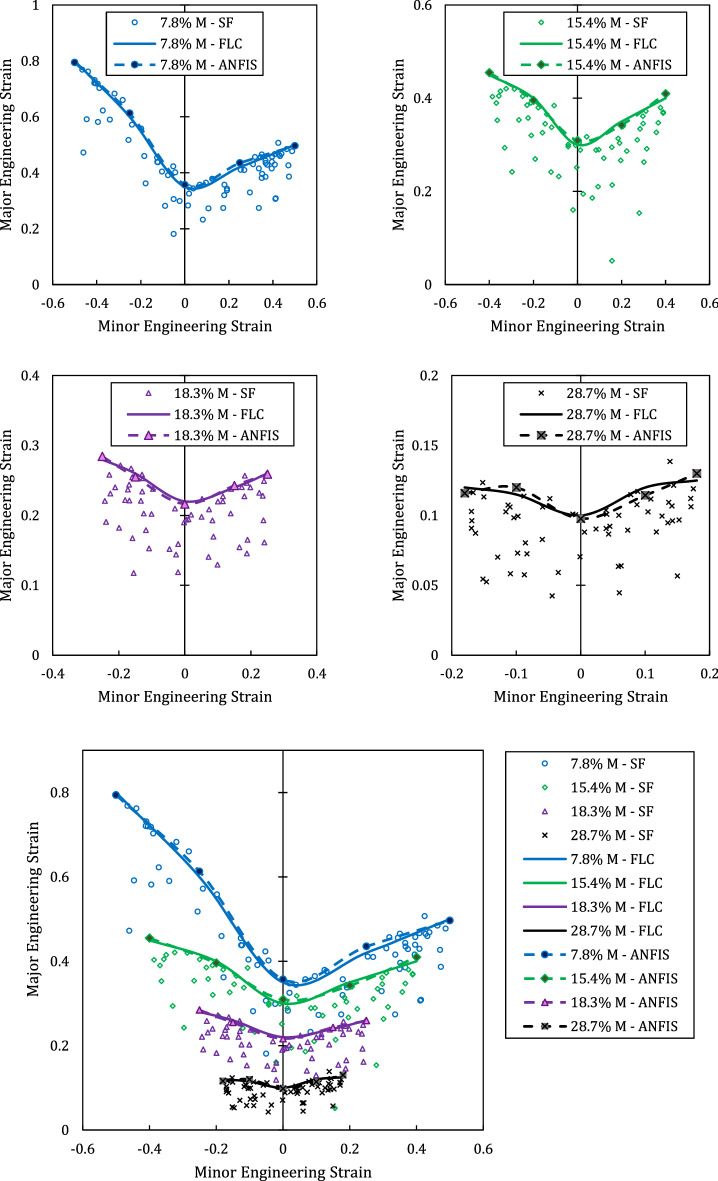
Actual experimental FLCs versus ANFIS-predicted curves for different martensite fraction conditions. These curves are utilized for training process.

In some point during training, the ANFIS network inevitably becomes over-fitted. To recognize this point, a parallel check called “validation” is performed. If the validation error value deviates from the training value, it means that the network is at the beginning of the over-fitting. As seen in Fig. 9b, until epoch 150, the difference between training and validation curves are small and they follow the approximately the same curve. At this point, the error of the validation process begins to deviate from training curve which is the sign of overfitting the ANFIS. Therefore, the ANFIS network at epoch 150 was saved with the error margin of 1.5%. At the next, the FLC predictions of ANFIS are presented. In Fig. 10, the predicted curves and actual curves of the selected samples for training and validation process are given. Since the data from these curves are used for training network, it is not surprising to observe very close predictions.

**Figure 11:**
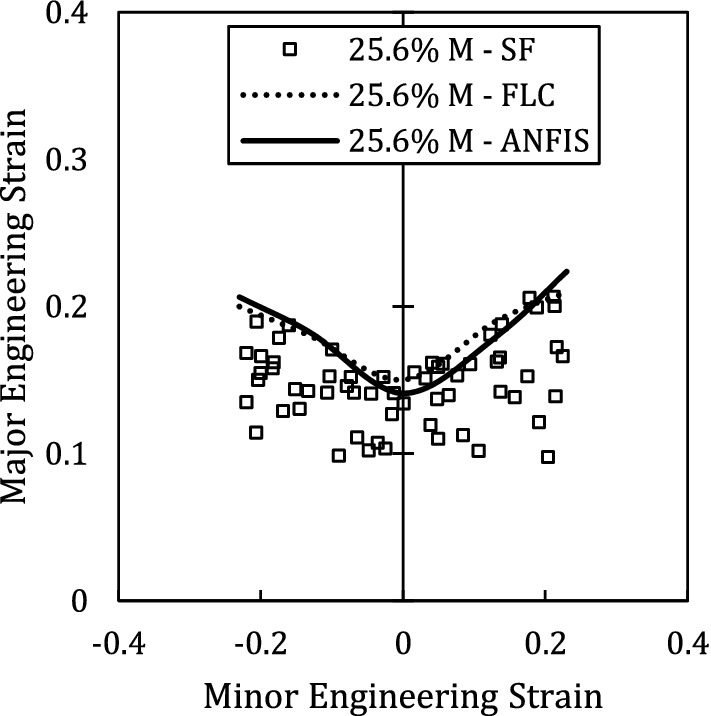
Schematic of ANFIS layers for two inputs and one outputs.

The condition of the last sample is unknown to the ANFIS model. Therefore, we test our trained ANFIS by feeding the martensite fraction of 25.6% sample to obtain the FLC. Figure 11 provides the FLC as predicted by ANFIS along with experimental FLC. The maximum error between the predicted value and experimental ones is 6.2% which is higher than the predicted values in training and validation process. However, this error is an acceptable error in comparison to other studies in which FLC is predicted by theoretical methods^37^.

## Conclusion

In industry, the influencing parameters on the formability are described in a linguistic form. For example, “large grain size decreases the formability” or “increase in cold work drops the FLC”. The inputs of ANFIS network, at the first step, divided into linguistic categories of, for example, low, medium and high. For different category, different rules are adopted by the network. Therefore, in industry this type of network could be very useful in term of both linguistic description of factors and also inclusion of several factors in the analysis. In this work, we tried to incorporate one of the major microstructural features of the austenitic stainless steels into consideration exploiting the capabilities of ANFIS. The amount of stress-induces martensite in the 316 is a direct effect of the cold work in these sheets. Using experimental and ANFIS analyses, it was determined that increase in the martensite fraction in this type of austenitic stainless steels results in significant drop in the FLC of 316 sheets such that increase from 7.8 to 28.7% of martensite fraction drops FLD0 from 0.35 to 0.1, respectively. On the other hand, the trained and validated ANFIS network using 80% of available experimental data could predict the FLC with maximum 6.5% error which is an acceptable error margin in comparison to other theoretical procedures and phenomenological relations.

## Data Availability

The datasets used and/or analyzed during the current study available from the corresponding author on reasonable request.
